# Robust performance of a novel stool DNA test of methylated *SDC2* for colorectal cancer detection: a multicenter clinical study

**DOI:** 10.1186/s13148-020-00954-x

**Published:** 2020-10-30

**Authors:** Jianping Wang, Side Liu, Hui Wang, Lei Zheng, Changchun Zhou, Guoxin Li, Rongkang Huang, Huaiming Wang, Chujun Li, Xinjuan Fan, Xinhui Fu, Xinying Wang, Hongliang Guo, Jie Guan, Yanlai Sun, Xilin Song, Zengjun Li, Dianbin Mu, Jujie Sun, Xianglin Liu, Yan Qi, Feng Niu, Chunhua Chen, Xiaolin Wu, Xianshu Wang, Xianrang Song, Hongzhi Zou

**Affiliations:** 1 Department of Colorectal Surgery, Guangdong Institute of Gastroenterology, Guangdong Provincial Key Laboratory of Colorectal and Pelvic Floor Diseases, The Sixth Affiliated Hospital, , Sun Yat-Sen University, Yuancun Erheng Road, Guangzhou, 510655 Guangdong China; 2 Department of Gastroenterology, Nanfang Hospital, Southern Medical University, 1838 Guangzhou Avenue North, Guangzhou, Guangdong China; 3 Clinical Laboratory, Nanfang Hospital, Southern Medical University, Guangzhou, Guangdong China; 4 Clinical Laboratory, Shandong Provincial Key Laboratory of Cancer Radiation, Shandong Cancer Hospital and Institute, Shandong First Medical University and Shandong Academy of Sciences, 440 Jiyan Road, Jinan, Shandong China; 5 Department of General Surgery, Nanfang Hospital, Southern Medical University, Guangzhou, Guangdong China; 6 Department of Gastrointestinal Endoscopy, The Sixth Affiliated Hospital, Sun Yat-Sen University, Guangzhou, Guangdong China; 7 Department of Pathology, The Sixth Affiliated Hospital, Sun Yat-Sen University, Guangzhou, Guangdong China; 8 Laboratory of Molecular Diagnostics, Department of Pathology, The Sixth Affiliated Hospital, Sun Yat-Sen University, Guangzhou, Guangdong China; 9 Department of Gastroenterology, Zhujiang Hospital, Southern Medical University, Guangzhou, Guangdong China; 10 Department of Surgery, Shandong Cancer Hospital and Institute, Shandong First Medical University and Shandong Academy of Sciences, Jinan, Shandong China; 11 Department of Endoscopy, Shandong Cancer Hospital and Institute, Shandong First Medical University and Shandong Academy of Sciences, Jinan, Shandong China; 12 Department of Pathology, Shandong Cancer Hospital and Institute, Shandong First Medical University and Shandong Academy of Sciences, Jinan, Shandong China; 13 Creative Biosciences (Guangzhou) CO., Ltd., Guangzhou, Guangdong China

**Keywords:** Colorectal cancer, Advanced adenoma, Stool DNA test, Methylation

## Abstract

**Background and Aims:**

Stool DNA testing is an emerging and attractive option for colorectal cancer (CRC) screening. We previously evaluated the feasibility of a stool DNA (sDNA) test of methylated *SDC2* for CRC detection. The aim of this study was to assess its performance in a multicenter clinical trial setting.

**Methods:**

Each participant was required to undergo a sDNA test and a reference colonoscopy. The sDNA test consists of quantitative assessment of methylation status of *SDC2* promoter. Results of real-time quantitative methylation-specific PCR were dichotomized as positive and negative, and the main evaluation indexes were sensitivity, specificity, and kappa value. All sDNA tests were performed and analyzed independently of colonoscopy.

**Results:**

Among the 1110 participants from three clinical sites analyzed, 359 and 38 were diagnosed, respectively, with CRC and advanced adenomas by colonoscopy. The sensitivity of the sDNA test was 301/359 (83.8%) for CRC, 16/38 (42.1%) for advanced adenomas, and 134/154 (87.0%) for early stage CRC (stage I–II). Detection rate did not vary significantly according to age, tumor location, differentiation, and TNM stage, except for gender. The follow-up testing of 40 postoperative patients with CRC returned negative results as their tumors had been surgically removed. The specificity of the sDNA test was 699/713 (98.0%), and unrelated cancers and diseases did not seem to interfere with the testing. The kappa value was 0.84, implying an excellent diagnostic consistency between the sDNA test and colonoscopy.

**Conclusion:**

Noninvasive sDNA test using methylated *SDC2* as the exclusive biomarker is a clinically viable and accurate CRC detection method.

**Chinese Clinical Trial Registry:**

Chi-CTR-TRC-1900026409, retrospectively registered on October 8, 2019; http://www.chictr.org.cn/edit.aspx?pid=43888&htm=4.

## Introduction

Colorectal cancer (CRC) ranks second in incidence and fifth in mortality among all cancers in China [1, 2]. The underlying neoplastic progression from adenoma to CRC endures up to 10 years, providing an extended window for CRC screening [3]. In the USA, the CRC mortality rate has declined by more than 50% over the last 40 years mainly as a result of increased population screening [4, 5]. In China, however, both CRC incidence and death rates have been continuously rising in recent years. Although screening for CRC by fecal occult blood test (FOBT) and colonoscopy has been widely performed for decades in China, it was only met with limited success [6–8]. The disappointing impact of conventional screening approaches on the reduction of CRC mortality in China suggests that a noninvasive, convenient, and more accurate strategy for early detection of CRC is clearly and urgently needed [9].

Stool DNA (sDNA) test is a novel method for screening colorectal neoplasms based on the fact that the colonic epithelial cells continuously shed into the gut lumen [10, 11]. The tumor cells excreted with stool can then be collected for isolation of DNA, in which aberrant genetic and epigenetic alterations can be detected. Through this approach, a mutant form of *KRAS* and aberrant methylation of certain genes including *BMP3*, *NDRG4*, *TFP12*, and *vimentin* were identified in colorectal neoplasms, some of which have been used as molecular markers to develop sensitive and user-friendly screening method [12–14]. Since the noninvasive nature, convenient sampling, and robust performance of sDNA testing are all desirable features for mass screening of average-risk population, the first such commercial product, Cologuard™ (Exact Sciences, Madison WI), was approved by US Food and Drug Administration for clinical use in 2014 [15]. In 2016, sDNA testing was recognized for the first time as a screening strategy by the US Preventive Services Task Force [16]. Since then, sDNA test has also been recommended in several CRC screening guidelines issued by the U.S. Multi-Society Task Force, National Comprehensive Cancer Network, and American Cancer Society [17–19].

*SDC2* belongs to syndecan family and encodes an integral membrane protein that is heavily glycosylated to act as a receptor for extracellular matrix components. It has been reported to play a critical role either as a tumor suppressor, such as in osteosarcoma [20], or an oncogene, promoting survival and metastases in breast cancer [21]. Hypermethylation of *SDC2* promoter region is a frequent epigenetic change taking place during the development of colorectal neoplasms and has been successfully detected in several types of clinical specimens including tissue, stool, and serum samples [22–26]. More importantly, the methylation test based on fecal samples can detect methylated *SDC2* in a large number of early stage CRC and advanced adenomas, making it an optimal target for developing a novel diagnostic marker for early detection [25]. In our previous pilot study, the methylation status of *SDC2* was evaluated in CRC cell lines and tissues as well as stool samples from CRC patients and healthy individuals to show that methylated *SDC2* might be a valuable biomarker in CRC detection [27]. Subsequently, we developed a brand-new sDNA test kit (Colosafe) using methylated *SDC2* as the exclusive marker for detection of colorectal neoplasms and conducted a clinical trial within three hospitals to evaluate its performance. The product was soon approved by National Medical Products Administration (NMPA) in November 2018 and became the first commercially available sDNA testing product on the market in China [28].

We hereby report the outcome of the multicenter clinical trial conducted to evaluate the effectiveness and accuracy of the sDNA test kit in a large case–control cohort for the fecal detection of CRCs and advanced adenomas. In addition, we tested patients with unrelated cancers and disorders to further examine its specific detection of CRC rather than a variety of interfering diseases.

## Methods

### Study design

The clinical trial was designed as a case–control study conducted within three hospitals, which were parallel-controlled, and enrolled over 1000 participants following the Technical Guidelines for Clinical Trials of in vitro Diagnostic Reagents released in 2014 by NMPA. The trial registration number at the Chinese Clinical Trial Registry, a World Health Organization (WHO) International Clinical Trial Registry Platform register, is Chi-CTR-TRC-1900026409. The study was approved by the Institutional Review Board (IRB) at each of the three hospitals, The Sixth Affiliated Hospital of Sun Yat-sen University (IRB No.: C2017001), Nanfang Hospital of Southern Medical University (IRB No.: NFEC-201705-Q1), and Shandong Cancer Hospital and Institute (IRB No.: SDZLEC2017-021). From June 2017 to July 2018, participants were enrolled at these three tertiary care units, and written informed consent was obtained from all participants who were eligible for final analysis. Study design, data collection, and statistical analysis were monitored by Beijing Jyton-Kannel Medical Tech. Co., Ltd., a contract research organization. The primary evaluation indexes include sensitivity, specificity, and Kappa value to assess the detecting accuracy of the sDNA test for CRC and the diagnostic consistency between the sDNA test and colonoscopy.

### Study population

Target population was enrolled including patients with definitive or suspected diagnosis of CRC, asymptomatic persons, individuals carrying gastroenterological benign lesions such as hemorrhoids and polyps, healthy normal controls, and subjects with other types of cancers. There was no age restriction on the cohort and almost equal number of male and female participants. The CRC patients were recruited at no less than 30% of all the qualified participants. Participants who had routine medications of berberine, an interfering substance as previously reported [27], were excluded. Patients considered unsuitable for colonoscopy, such as those with pregnancy, hypertension, or heart diseases, as well as those who were unable or unwilling to provide written informed consent, were also excluded. Separately, one independent set with 40 individuals randomly selected from CRC patients across all three clinical sites was subjected for a postoperative follow-up study. Initially, 25 patients were enrolled from June 2017 to February 2018 into the postoperative group, which was supplemented with 15 more participants in July 2018 to bring the final number to 40. They were tested again within 3–8 months after resection during follow-up visits. Another independent set of 103 patients with various interfering diseases was also established and assessed using the sDNA test kit. All 103 patients were enrolled and tested at Shandong Cancer Hospital and Institute in July 2018. They did not undergo colonoscopy to follow the ethic rule of avoiding excessive medical care for patients in China.

### Clinical procedures

Every participant was required to provide 1–10 g (average 4.5 g) stool sample and to be examined by colonoscopy. Before bowel preparation for colonoscopy or after colonoscopy, fecal sample was collected in one of the three hospitals and sent to its medical testing laboratory according to the standard operation procedure. Only a unique product code on a stool collection device with no other identifiable information was provided to laboratory technicians for blinded testing. Colonoscopy combined with pathology report of tissue biopsy, the gold standard for diagnosis, was utilized to categorize each participant and for CRC staging. When a participant was diagnosed with two or more types of colorectal lesions, only the more or most advanced colorectal lesion would be used for classification. CRC was staged according to the 7th edition of Cancer Staging Manual from American Joint Committee on Cancer. Advanced adenomas were defined as the adenomas with either maximum diameter ≥ 1 cm, or high-grade intraepithelial neoplasia (HGIN), or substantial villous structures.

### Sample processing

All fecal samples were delivered locally, within 3 days of collection, to the designated medical testing laboratories at one of the three clinical sites. Stool specimens were immediately homogenized and centrifuged upon receipt. The supernatant was aliquoted and frozen at − 80 °C until further use. Frozen aliquots were subsequently tested in batches by laboratory technicians who had no prior knowledge of the results from colonoscopy.

### Target gene capture and bisulfite treatment

For each coded sample, 3.2 mL of stool supernatant was centrifuged in a solid phase extraction column embedded with polyvinylpolypyrrolidone after thawing. The target *SDC2* and the positive control β-actin (*ACTB*) were enriched and purified by a sequence-specific capture technology with some minor modifications [27]. Briefly, the centrifuged supernatant was added with guanidine isothiocyanate solution (Sigma) and sequence-specific oligonucleotide beads and was incubated at 95 °C for 15 min. After the solution was cooled at room temperature, the bead/hybrid capture complexes were washed twice using 1 × MOPS washing buffer, and then eluted out with 50 μL elution buffer. The eluate was then mixed with 100 μL sodium bisulfite, and the bisulfite-treated DNA was subsequently collected by magnetic beads. DNA/magnetic bead complexes were then washed twice using washing buffer and then denatured, and the DNA was eluted out from the beads with 60 μL nuclease-free water and used for amplification in the subsequent step. Positive and negative controls of *ACTB* were tested in parallel.

### Real-time quantitative methylation-specific PCR

Real-time quantitative methylation-specific PCR (qMSP) was employed to quantitatively detect *SDC2* and *ACTB* methylation status in stool samples. *ACTB* was amplified as a reference for DNA input. PCR reaction was prepared in a volume of 30 μL, containing 1 × Colorless GoTaq Flexi Buffer, 5 mmol/L MgCl_2_, 0.4 mmol/L dNTPs, 0.1 U/μL GoTaq Hot Start Polymerase (Promega), 0.5 μmol/L of each primer, 0.2 μmol/L probe, and 10 μL bisulfite-treated DNA from last step. PCR was performed in Roche LightCycler 480 II under the following cycling conditions: 95 °C for 5 min, 48 cycles of denaturation at 95 °C for 15 s, annealing at 58 °C for 30 s, and extension at 72 °C for 30 s, and a final cooling step at 40℃ for 30 s. Primers and probes in this study were identical to those reported in our previous study [27].

### Interpretation and data analysis of real-time qMSP of *SDC2*

Abs Quant/2nd Derivative Max method in Roche LightCycler 480 II (Roche, Basel, Switzerland) was used to calculate cycling threshold (CT value) by assigning a prespecified cut-off value for each amplification curve. Every batch of PCR reactions were performed with three controls, an *ACTB* internal control, methylated *SDC2* as a positive control, and unmethylated *SDC2* as a negative control. If a sample showed no amplification of methylated *SDC2*, no CT value would be assigned for the sample. All valid samples should satisfy the requirement of CT value of *ACTB* ≤ 36. If a sample has CT value of *ACTB* > 36, the result would be considered invalid. Target gene capture, bisulfite treatment, and PCR amplification would be rerun using a second aliquot from the sample. The CT threshold of 38 was selected to dichotomize the result of qMSP for methylated *SDC2* mainly to maximize sensitivity and minimize false positive rate. Therefore, stool samples with CT values ≤ 38 for *SDC2* methylation were called “positive” and were most likely associated with CRC and advanced adenomas. In contrast, stool samples with CT value > 38 or no CT value assigned were reported negative and were unlikely associated with advanced colorectal neoplasia. All negative samples without CT values assigned from Roche LightCycler 480 II would be arbitrarily given a value of 43 each in order to quantitatively compare methylation levels between CRCs, advanced adenomas, and normal controls.

### Statistical analysis

Sensitivity and specificity with 95% confidence interval (95% CI) were used to assess the accuracy of the sDNA kit. As detection of advanced colorectal neoplasia (CRC plus advanced adenoma) was the primary goal in screening setting, patients who had clinical findings other than advanced colorectal neoplasia including benign polyps and negative findings on colonoscopy were all grouped together and classified as normal subjects to calculate specificity. Kappa value was used to assess the diagnostic consistency between the sDNA test and colonoscopy. Kappa value > 0.75 suggests that the test has a substantial diagnostic consistency. ROC curve was constructed to evaluate diagnostic performance of the test. Pearson Chi-square test was employed to compare the qualitative methylation levels and clinicopathological features of CRC patients. Additionally, Kruskal–Wallis test was performed to compare the quantitative methylation levels among CRCs, advanced adenomas, and normal cases. *P* < 0.05 was considered statistically significant. All analyses were conducted with SPSS 20.0 (IBM Corporation, Armonk, NY, USA).

## Results

### Selection of hospital-based study cohorts for sDNA testing after series of exclusions

**Fig. 1:**
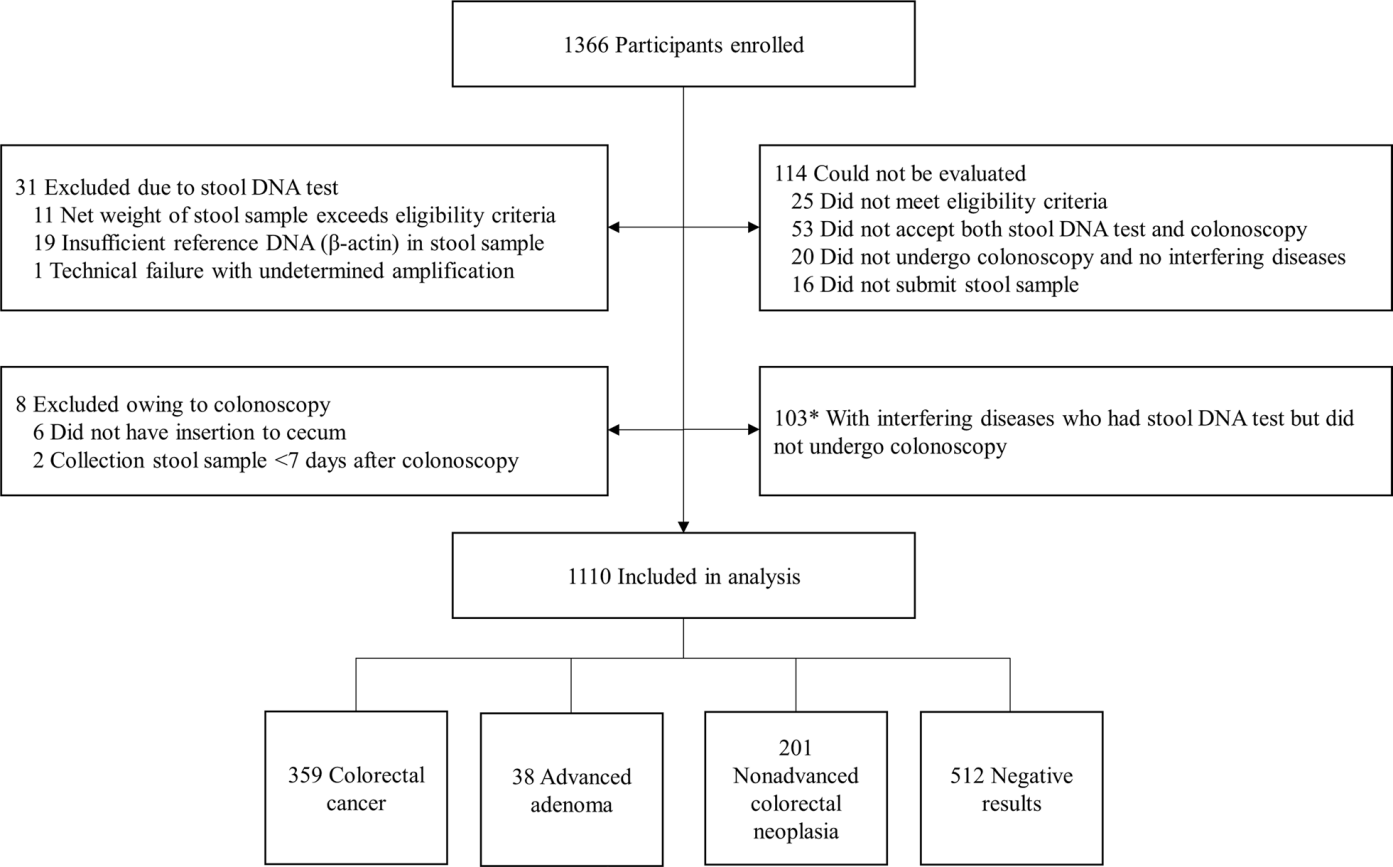
The flowchart of study design. * An independent set of 103 patients discussed in the manuscript

A total of 1366 participants were enrolled at three hospitals, and 1110 of them who completed both sDNA test and colonoscopy (81.3%) were subjected to final analysis after excluding 153 (11.2%) individuals who could not be evaluated and another set of 103 (7.5%) patients with interfering diseases who took sDNA test but did not have colonoscopy (Fig. 1). The exclusion of 153 enrolled subjects was due to a variety of reasons. A total of 31 subjects were excluded as a result of invalid stool DNA tests, arising from excessive stool specimens (*n* = 11), insufficient reference DNA (*n* = 19), and failed gene amplification (*n* = 1). Another group of 114 individuals were not analyzed because 25 did not meet all the eligibility criteria, 53 did not have both sDNA test and colonoscopy results, 20 without interfering diseases did not undergo colonoscopy examinations, and 16 did not submit their stool samples for SDC2 methylation test. Finally, 8 more patients were not evaluated since their colonoscopy examinations were disqualified due to incomplete procedure (*n* = 6) and erroneous post-colonoscopy sample collection (*n* = 2) (Fig. 1). For all three aforementioned categories of 1110, 153, and 103 participants, demographic characteristics including gender and age groups of all enrolled participants are provided in Table 1. The main group of 1110 participants was further classified as CRC (*n* = 359), advanced adenomas (*n* = 38), nonadvanced colorectal neoplasia (*n* = 201), and negative findings (*n* = 512), based on colonoscopy and pathology reports from tissue biopsies (Fig. 1). These participants were relatively evenly distributed through three clinical sites, each of which has at least 30% of the total cohort, but the independent set of 103 patients was solely enrolled at Shandong Cancer Hospital and Institute to assess the effect of a plethora of digestive and other diseases on fecal level of *SDC2* methylation (Additional file 1: Supplementary Table S1). To be noted, subjects diagnosed with nonadvanced colorectal neoplasia (*n* = 201) and negative findings on colonoscopy (*n* = 512) are combined and treated as “normal” controls (*n* = 713) in calculating sensitivity and specificity of the sDNA test.

**Table 1:** Demographic characteristics of the study participants

Characteristic	Subjects included for analysis (*N* = 1110) (%)	Subjects excluded for analysis (*N* = 153) (%)	Subjects with interfering diseases (*N* = 103) (%)
Gender-*no.* (%)			
Male	569 (51.3)	63 (41.2)	76 (73.8)
Female	541 (48.7)	90 (58.8)	27 (26.2)
Age year-*no.* (%)			
≦ 39	83 (7.5)	17 (11.1)	5 (4.9)
40–49	288 (25.9)	37 (24.2)	11 (10.7)
50–59	387 (34.9)	51 (33.3)	35 (34.0)
60–69	267 (24.1)	40 (26.1)	36 (35.0)
≧ 70	85 (7.7)	8 (5.2)	16 (15.5)
Mean ± SD	54.1 ± 11.2	53.5 ± 10.8	58.7 ± 10.7

### Performance of the sDNA Test in detecting CRC and advanced adenomas

**Fig. 2:**
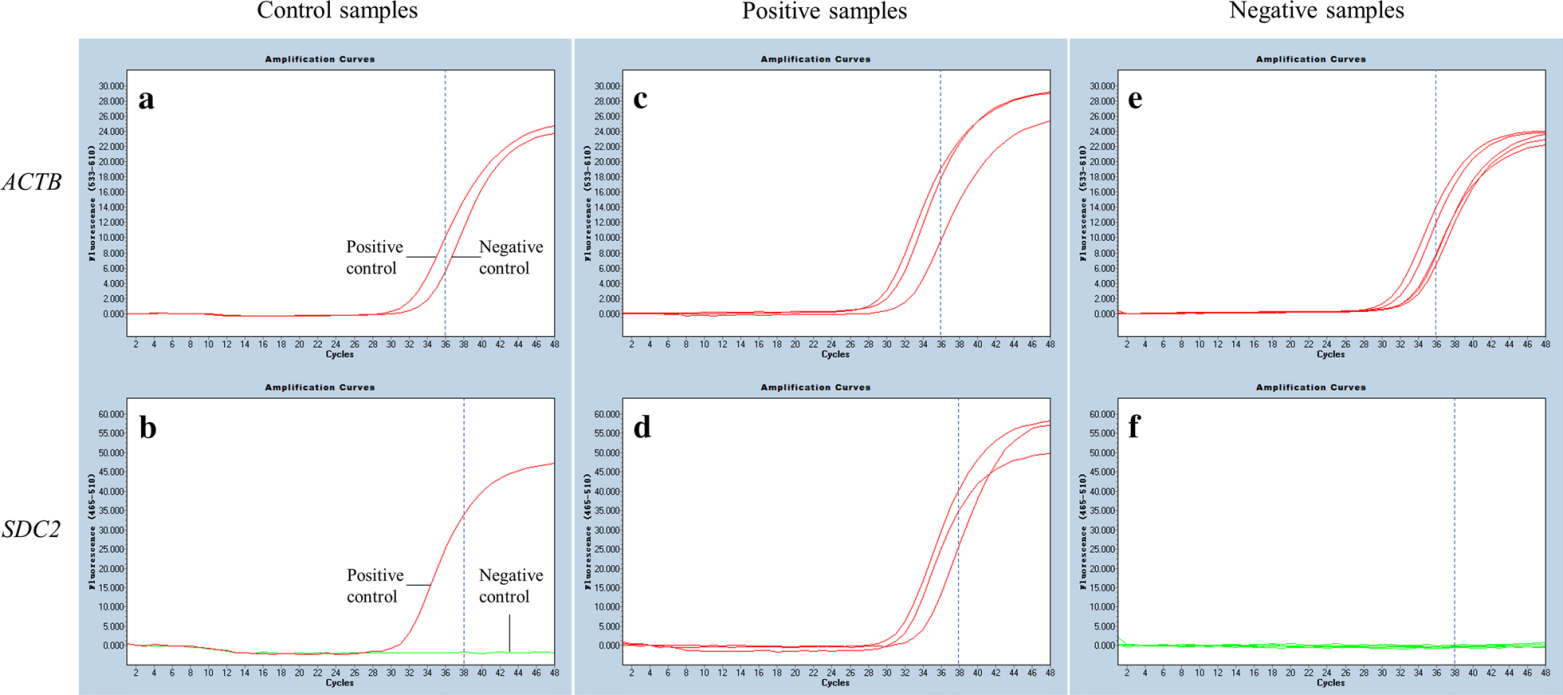
Amplification by real-time qMSP of *ACTB* control, methylated, and unmethylated *SDC2*

**Fig. 3:**
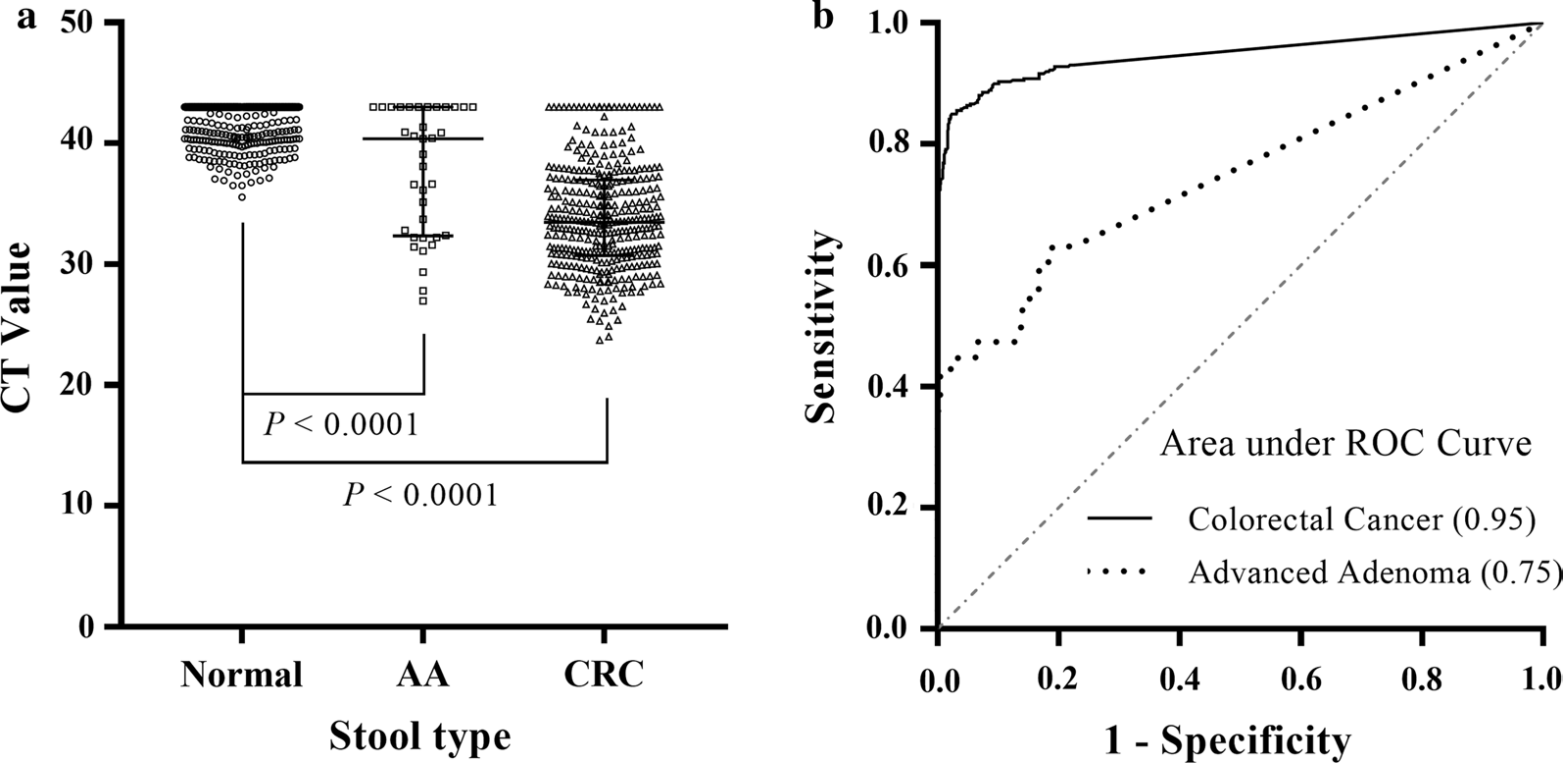
**a** Levels of methylated *SDC2* in 713 normals, 38 advanced adenomas (AA), and 359 CRCs. **b** ROC curves of the sDNA testing of *SDC2* methylation for the detection of CRC and advanced adenomas

We have previously demonstrated that higher fecal methylation levels of *SDC2* were detected in adenomas and CRCs than in normal controls by quantifying with qMSP [27]. In our current clinical trial, a total of 1110 stool samples from 359 CRC and 38 advanced adenoma patients vis-a-vis 713 normal control subjects quantified by qMSP returned valid results with CT value of *ACTB* ≤ 36 (Fig. 2a, c, e). For every batch of qMSP reactions, the positive control of methylated *SDC2* always displayed CT value ≤ 38, along with a large proportion of the CRC and advanced adenoma cases (Fig. 2b, d, red curves), while the negative control of unmethylated *SDC2,* as well as almost all of the normal control samples, exhibited no amplification even after 48 cycles of PCR (Fig. 2b, f, green baselines). Median CT values, which correspond inversely to *SDC2* methylation levels, were, respectively, 33.46 (30.73, 36.99) for CRCs (*n* = 359), 40.41 (32.35, 43.00) for adenomas (*n* = 38), and 43.00 (43.00, 43.00) for normal controls (*n* = 713, *P* < 0.0001; Fig. 3a). Additionally, area under ROC curve (AUC) value of the sDNA test was 0.95 (95% CI 0.93–0.97) for CRC detection, indicating that the test had excellent performance at discriminating CRCs versus non-neoplastic polyps and negative findings (Fig. 3b). For advanced adenomas, the AUC value decreased to 0.75 (95% CI 0.67–0.86), showing that the test’s performance was only fair at detecting the precancerous lesions (Fig. 3b).

The sDNA test was able to identify 301 out of 359 CRC cases with a sensitivity of 83.8% (95% CI 79.5–87.4) (Table 2). For 154 cases whose stage I or II tumors were confined to bowel walls, the sensitivity was even higher at 87.0% (95% CI 80.4–91.7) than those of all CRC, advanced colorectal neoplasia, stage III, IV, or I-III CRC combined (Table 2, Additional file 1: Supplementary Figure S1). Detection rate did not vary significantly according to patient’s age, tumor location, differentiation, and TNM stage, except for gender (*P* = 0.041) (Table 3). The sensitivity did decline significantly to 42.1% (95% CI 26.7–59.1) for detecting advanced adenomas. These detectable precancerous lesions not only include large size adenomas (≥ 1 cm, 21/38) but also adenomas with substantial villous structures (≥ 25%) and HGINs (17/38), polyps highly likely to progress further to CRCs (Additional file 1: Supplementary Table S2). When CRC and advanced adenomas were combined, the sDNA test detected 79.9% (95% CI 75.5–83.6) of the 397 cases of advanced colorectal neoplasia (Table 2). The specificity of the sDNA test was 98.0% (95% CI 96.6–98.9) in 713 participants who had findings other than CRC or advanced adenoma (e.g., nonadvanced colorectal neoplasia, non-neoplastic findings and negative results on colonoscopy) (Table 2). The kappa value for CRC detection was 0.84 (95% CI 0.81–0.88), representing an excellent diagnostic consistency with colonoscopy (Table 2). Notably, for early stage cancers (I-II), the kappa value was slightly higher at 0.86 (95% CI 0.82–0.90). For the detection of advanced adenomas alone, kappa value was considerably lower at 0.45 (95% CI 0.28–0.59), indicating that the diagnoses from the sDNA testing were in moderate agreement with those of colonoscopy. However, if CRC and advanced adenomas were combined, kappa value was 0.81 (95% CI 0.76–0.85), showing that the sDNA test could still be highly reliable for detecting advanced colorectal neoplasia (Table 2). Separately, we also evaluated these performance characteristics for each one of the three participating centers and found little variation between them (Additional file 1: Supplementary Table S3), which could possibly be attributed to standardized sample collection, optimized testing procedure, and enrichment of CRC cases.

**Table 2:** Performance characteristics of the sDNA test for the detection of colorectal neoplasia

Category	Colonoscopy (*N* = 1110) *no*	Stool DNA test (*N* = 1110)		
		Positive (*n* = 331) *no*	Sensitivity (95% CI) %	Kappa (95% CI)
Colorectal cancer				
Any	359	301	83.8 (79.5–87.4)	0.84 (0.81–0.88)
Stage I–II	154	134	87.0 (80.4–91.7)	0.86 (0.82–0.90)
Advanced adenoma	38	16	42.1 (26.7–59.1)	0.45 (0.28–0.59)
Advanced colorectal neoplasia^a^	397	317	79.9 (75.5–83.6)	0.81 (0.76–0.85)

			Specificity (95% CI)	
Normal^b^	713	14	98.0 (96.6–98.9)	N/A

^a^Advanced colorectal neoplasia includes colorectal cancer and advanced adenoma

^b^Nonadvanced colorectal neoplasia, non-neoplastic findings and negative results on colonoscopy

**Table 3:** Association of *SDC2* methylation with clinicopathological features of CRC

Variable	Colonoscopy (*N* = 359) *no*	Stool DNA test positive (*N* = 301) *no.* (*n/N*, %)	Stool DNA test negative (*N* = 58) *no.* (*n/N*, %)	*P* value*
Gender				
Male	234	203 (86.8)	31 (13.2)	**0.041#**
Female	125	98 (78.4)	27 (21.6)	
Age				
≦ 49	83	66 (79.5)	17 (20.5)	0.500
50–59	104	86 (82.7)	18 (17.3)	
60–69	111	97 (87.4)	14 (12.6)	
≧ 70	61	52 (85.2)	9 (14.8)	
TNM stage				
I	46	40 (87.0)	6 (13.0)	0.323
II	108	94 (87.0)	14 (13.0)	
III	160	133 (83.1)	27 (16.9)	
IV	45	34 (75.6)	11 (24.4)	
Location 1				
Proximal	50	38 (76.0)	12 (24.0)	0.104
Distal	309	263 (85.1)	46 (14.9)	
Location 2				
Colon	127	100 (78.7)	27 (21.3)	0.052
Rectum	232	201 (86.6)	31 (13.4)	
Differentiation				
Well	44	38 (86.4)	6 (13.6)	0.641
Moderate	257	213 (82.9)	44 (17.1)	
Poor	27	24 (88.9)	3 (11.1)	

^*^P value was calculated by Pearson Chi-Square Test

^#^Bold indicates *P* < 0.05

### The sDNA test performance in postoperative patients and interfering diseases

The veracity of the sDNA test was further examined in 40 individuals randomly selected from 359 CRC patients who had undergone surgeries to remove their colorectal tumors. All group members were diagnosed by colonoscopy with stage I, II, or III CRC except for one stage IV cancer and with tumors localized across the colon and rectum. The age of these individuals ranges from 41 to 75, and there were more males than females (23 vs 17) (Additional file 1: Supplementary Table S4). Using the sDNA test kit, higher *SDC2* methylation levels were detected in 38 of the 40 CRC patients with 2 false negatives before colectomies. After tumor resection, all of them were tested negative except for one patient whose result was nullified due to his invalid assay value of *ACTB* internal control (Additional file 1: Supplementary Table S4). In conclusion, 37 CRC patients, who were tested positive before surgeries, were tested negative after their colorectal tumors were removed.

To further investigate the specificity of the sDNA test, a total of 103 patients who had interfering diseases but no symptoms of advanced colorectal neoplasia were recruited. These interfering diseases included a plethora of cancers and other types of ailments, in which 51/103 (49.5%) were located in the digestive tracts, and 52/103 (50.5%) were originated in other parts of the body (Additional file 1: Supplementary Table S5). The ailments located in the digestive tracts included 30 cancers and 21 inflammatory diseases. Esophageal and gastric cancers (*n* = 12 and 11, respectively) were the two major types with more than two-thirds of the cancer cases while gastritis and esophagitis (*n* = 11 and 8, respectively) accounted for almost all the inflammatory diseases. For interfering diseases outside the digestive tracts, we focused on lung cancer (*n* = 14), prostate cancer (*n* = 10), rheumatoid arthritis (*n* = 8), and microbial infections (*n* = 10) to examine their effects on the specificity of the sDNA test. The age range of the whole independent set was from 31 to 85, with average at 59, and the majority of the participants were males (76/103). A total of 101 patients were tested negative in *SDC2* methylation. Among them, 50 patients had cancers or inflammation within the digestive tracts while 51 others had other types of cancers and disorders that were not of gastrointestinal origin (Additional file 1: Supplementary Table S5). For two patients who had positive test results, one was diagnosed with lung cancer and the other gastritis. The specificity for this set is 98.1%, almost identical to that of the normal control group of 713 subjects (Table 2, Additional file 1: Supplementary Table S5). Apparently, the interfering diseases, in particular cancers in the upper digestive tracts and inflammatory bowel disease, exhibit minimal effect on the level of fecal methylated *SDC2*, and hence the false positive rate of the sDNA testing.

## Discussion

Earlier detection through CRC screening is strongly associated with favorable prognosis as 5-year survival rate reaches as high as 90% when the malignancy is still diagnosed at localized stage [5]. Hence, it is of paramount importance to develop a fecal DNA methylation test that is sensitive to detect early-stage CRC and precancerous lesions for effective surgical and therapeutic interventions. In the current multicenter clinical study, our newly developed sDNA test demonstrated an overall sensitivity for all CRCs at 84%, which is further improved to 87% for stage I and II cancers combined. Such performance is quite robust for a single-target test and comparable to 90% sensitivity reported by Han et al. in a recent clinical trial of a cohort of 585 Koreans using an independently developed stool-based *SDC2* methylation test [29]. Our sDNA test can also detect 42% of all the advanced adenoma cases, a sensitivity that is more or less in agreement with results from our previous study [27] and other groups [13, 14, 29], supporting the notion that promoter hypermethylation of *SDC2* is a frequent and early event in the colorectal normal-adenoma-carcinoma sequence. All performance characteristics including sensitivity, specificity, and kappa value did not vary significantly across the three participating centers where the stool specimens were collected and the tests were independently performed, indicating that the trial is sufficiently powered at each site and adequately parallel-controlled to minimize factors that can affect test consistency. Furthermore, our sDNA test’s capability of detecting CRC, like Cologuard’s, is not affected by its TNM stage, location, and differentiated state, ensuring that the detection for tumors still at early stage, in proximal colon, or with poor differentiation is as equally sensitive as for those that are at late stage, in distal colon, or well differentiated, all clinically desirable characteristics of a diagnostic test for CRC [14, 27]. Altogether, these data highlight *SDC2* as a highly valuable biomarker, and the simple and affordable single-target sDNA test based on methylated *SDC2* is capable of detecting early CRC with substantial accuracy.

A reliable and accurate screening approach combines superior sensitivity with highest specificity possible. The high sensitivity of *SDC2* methylation test achieved for CRC detection can be attributed to the extremely high frequency of marker’s promoter hypermethylation in tumor tissues [22]. In The Cancer Genome Atlas (TCGA) database, profiling data on a total of 45 pairs of CRC tumor tissues and adjacent normal epithelia by Infinium Human Methylation 450 K Beadchip arrays (Illumina, San Diego, CA) revealed that hypermethylation in *SDC2* promoter region occurred in 93% (42/45) of the tumor tissues (unpublished data). Consistently, Niu and colleagues detected in tissue specimens a very high frequency of *SDC2* promoter hypermethylation in 83% of the CRC tumors (103/124) and further demonstrated that methylation level was higher in 96.8% (120/124) of cancers than in their paired adjacent normal epithelia [27]. In addition, Bartak et al. confirmed previous published findings by showing that 87% (13/15) of the colonic tumor tissues had promoter hypermethylation in *SDC2* gene by bisulfite pyrosequencing and detected its methylation in 89% of CRC in plasma specimens, a sensitivity similar to that from Oh et al. (87%) in serum samples and ours (84%) in stools [24, 25, 27]. The false negative rate consistently stood at 10–15% when fecal samples were used for CRC detection [27, 29]. For the false negative stool samples in our clinical trial, we did sequence the targeted region of *SDC2* gene using isolated DNA by sequence-specific capture from fecal specimens to confirm that no methylation had taken place (unpublished data). However, we did not examine the methylation status in the original tumor or adenomatous tissues as well as adjacent normal controls by bisulfite pyrosequencing.

Lower specificity of a cancer-screening test leads to more false positives in a population screening as a result of low cancer prevalence. In the current study, we were encouraged by the exceptional specificity of 98.0% that our test achieved for CRC detection, a desirable feature for a future screening test. It is significantly higher than most of the hitherto-published specificity values for stool-based DNA tests, some of which also used methylated *SDC2* as the diagnostic marker [12, 14, 25, 29]. There are several possible and reasonable explanations for the visibly large discrepancy. First, our sDNA test employs a single methylation marker for fecal detection of CRC while the multitarget stool-based DNA test [14] screens mutations in *KRAS*, assays for hypermethylated promoters of *BMP3* and *NDRG4*, and measures amount of hemoglobin in stool. The latter approach enhanced its sensitivity by assessing multiple molecular targets but sacrificed its specificity by piling up false positive rate of each single marker. Second, our sDNA test retrieves target DNA by a sequence-specific capture technology to effectively eliminate background noise from massive amounts of contaminating plant, animal, and bacterial genomic DNA in qMSP assays, which is a more specific method than column-based affinity purification or conventional phenol–chloroform extraction [25, 29]. Third, the different primer and molecular probe sets as well as assay conditions independently designed and developed by the two research teams [27, 29] are also potential contributors to the varied specificity. Fourth, due to the minuscule amount of human DNA in stool, Han’s group conducted two rounds of PCR of 35 and 40 cycles, respectively, with the first one to enrich *SDC2* target and the second one to detect methylated *SDC2*. Additionally, the qMSP was performed in duplicates and called positive if only one of the two reactions showed CT value ≤ 40. Such extreme measures did increase the sensitivity of their test but in the meantime probably lowered its specificity in normal healthy controls. Fifth, using a quantitative sample collection device, our sDNA test draws on average 4.5 g of stool sample from a single spot on a shaped stool rather than whole stools [14] or a mixture from multiple spots [29]. Even though we showed that the different sample collection strategies did not seem to alter the false positive rate in a group of 20 normal controls in our pilot study [27], similar tests were not performed and reported in other studies [14, 29]. Nevertheless, no matter what the aforementioned factors actually account for the discrepancy, the superior specificity provides room for further improvement of our sDNA test by means of supplementing additional methylation markers to drastically boost its sensitivity and simultaneously maintain its high specificity. As Cologuard has become a more and more popular screening choice in the USA [16–19], Colosafe may offer a promising and affordable alternative in CRC screening for the huge Chinese population.

As an extension of the validation study of the sDNA test, its performance was further examined in a total of 40 randomly selected postoperative patients diagnosed with CRC. All CRC patients, who had been originally tested positive of *SDC2* methylation, were tested negative after tumor resection. The data strongly suggests that the original positive results were due to the exfoliated cells from those tumors that had been removed. The study outcome of postoperative patients is similar to what has been found by Kisiel and his colleagues, who reported that the sDNA testing using methylation markers was an informative screening approach that might be useful in cancer surveillance [30]. Since the local recurrence of CRC after curable surgeries often accounts for more than 50% of overall recurrent cases [31], the sDNA test can serve as an alternative option to monitor postoperative patients to reduce physical trauma and emotional stress during their compulsive follow-up care.

*SDC2* methylation level has been found to elevate in tumor tissues of some types of cancers such as glioma and gastric cancer [32, 33]. In their clinical trial of a *SDC2* methylation test in detecting CRC, Han et al. also recruited 23 patients with gastric cancer and 10 patients with liver cancer and showed that 30.4% of gastric cancer patients (7/23) and 30% liver cancer patients (3/10) were tested positive for *SDC2* methylation [29]. Additionally, stool DNA testing was also used for the detection of pancreatic cancers albeit with different candidate methylation markers [34]. Hence, it is crucial to define the effects of interfering diseases, in particular the malignancies of the digestive tract including esophageal, gastric, pancreatic, and liver cancers, on the detection accuracy of fecal *SDC2* methylation test. In our study of an independent set of 103 patients who enrolled at the same clinical site and had unrelated cancers and diseases, the sDNA test again showed excellent specificity of 98.1% with only two exceptions. It did not detect *SDC2* methylation in any of the cancers in the digestive tract (0/30). This outcome is actually consistent with a 4-year follow-up study of a cohort of 1050 patients with false positive screening results from a multi-target stool DNA testing [35], in which Cotter and colleagues found that the incidence of new aerodigestive cancers in the false positive group did not exceed the estimate for the general population. Another smaller study of 30 patients with false positive results by Cooper et al. did not find non-colorectal lesions by second fecal DNA test, second colonoscopy, upper endoscopy, and thorough review of medical records 11 months after the initial test [36]. Taken together, these data suggest that the influence of interfering diseases may be minimal in a real-world practice. It is more likely that the false positive results were due to either false negative colonoscopy or occasional elevated methylation status in healthy individuals for some unknown reasons.

The sensitivity of the sDNA test for the detection of advanced adenomas was only half of that for CRC. Consequently, a large proportion of these cases were false negatives, raising concerns that it would result in more missed diagnoses and delayed interventions than colonoscopy. There are a total of 22 advanced adenomas with negative results for SDC2 methylation. Among them, 41% (9/22) has HGIN compared with 88% (14/16) for 16 advanced adenomas with positive results (Additional file 1: Table S2), and the difference is statistically significant (*P* = 0.002). Hence, these false negatives are associated with less HGIN, implying that they may be less progressed than those with positive results. However, the statistical analysis is not adjusted for the effects of tumor size and villous components on the outcome, and additional studies in large, independent sample sets are needed to find out whether such correlation can be validated. For an individual with negative test result, it is recommended that he or she repeats the sDNA test within 1–3 years to reduce chance of missing diagnosis of a lesion in progression.

In conclusion, we have demonstrated, in our multicenter clinical trial, admirable performance characteristics of a novel single-target sDNA test, potentially a promising and alternative option for CRC screening. However, there are still certain limitations associated with our current investigation. First, the robust performance of the sDNA test in this hospital-based cohort is not guaranteed to replicate in a mass screening when it is used as a first-line choice, and future large-scale validation study is required to accurately assess its effectiveness in an average-risk population. Second, the evaluation of the sDNA test was not performed in head-to-head comparison to FIT, another stool-based routine screening strategy for CRC. Whether the *SDC2* methylation test outperforms its appropriate competitor needs to be further addressed in a representative screening setting. Third, the number of cases of advanced adenomas, in particular the pathology information regarding villous and serrated adenomas, is limited, hence lacking sufficient power to accurately estimate the test’s sensitivity and to perform further covariate analysis of these precancerous lesions. Fourth, although the effect of a plethora of interfering diseases on the sDNA test was assessed, none of them was tested in significant number of patients to reach statistically meaningful conclusion. Fifth, whether the sDNA test will retain its robust performance in other global ethnic groups is unknown. Although methylated *SDC2* is a successful CRC biomarker for Chinese and Koreans, and qMSP outcome seems immune to interfering effects of various substances from diets [27, 29], pilot trials are still warranted for other ethnic groups to ensure a comparable performance of the test before its intended use en masse. Sixth, despite delivering strong performance and substantial accuracy, the sDNA test still has room to improve, particularly its sensitivity to detect CRC and advanced adenomas. Recently, more DNA methylation markers have been identified for fecal detection of CRC and advanced adenoma with increased sensitivity [37], paving the way to generate enhanced product for better prevention of this malignant disease.

## Supplementary information

**Additional file 1.** Supplementary tables and figures.

## Data Availability

The datasets analyzed during this study are available from the corresponding author upon reasonable request.

## Abbreviations

CRC: Colorectal cancer
sDNA: Stool DNA
FOBT: Fecal occult blood test
FIT: Fecal immunochemical test
CI: Confidence interval
CT: Cycling threshold
NMPA: National Medical Products Administration
qMSP: Quantitative methylation-specific PCR
ROC: Receiver operation curve
AUC: Area under ROC curve
