# Supplementary material for: Robust performance of a novel stool DNA test of methylated SDC2 for colorectal cancer detection: a multicenter clinical study
Source: Clin Epigenetics. 2020 Oct 30;12:162. doi: 10.1186/s13148-020-00954-x (PMC7602331; doi:10.1186/s13148-020-00954-x)
Supplement: Supplementary file 1 — Additional file 1. Supplementary tables and figures. [file 13148_2020_954_MOESM1_ESM.docm]

**Supplementary Tables and Figures**

Table S1. Cases included and excluded in three clinical sites.

| Hospital | Subjects included for analysis (N=1110) (%) | Subjects excluded for analysis (N=153) (%) | Subjects with interfering diseases (N=103) (%) |
| --- | --- | --- | --- |
| The Sixth Affiliated Hospital of Sun Yat-sen University | 409 (36.8) | 46 (30.1) | 0 (0) |
| Nanfang Hospital of Southern Medical University | 367 (33.1) | 73 (47.7) | 0 (0) |
| Shandong Cancer Hospital and Institute | 334 (30.1) | 34 (22.2) | 103 (100) |

Table S2. Clinical and pathological features of patients with advanced adenomas

| ID | Age | Gender | Test result (+/−) | Villous  (Y/N) | Serrated  (Y/N) | Location | Size (cm) | Pathology |
| --- | --- | --- | --- | --- | --- | --- | --- | --- |
| 1084 | 33 | female | − | N | N | DC | 1 | TA |
| 1133 | 70 | male | − | Y | N | AC | 2.5 | VA, TA |
| 1146 | 65 | female | + | N | N | Rectum | 0.3-0.4 | HGIN |
| 1165 | 70 | male | − | Y | N | Rectum | NA | VA, TA, HGIN |
| 1167 | 57 | male | + | N | N | Rectum | 0.2-0.3 | HGIN |
| 1195 | 50 | male | + | N | N | Rectum | 0.2-0.4 | HGIN |
| 1215 | 78 | male | + | Y | N | Rectum | 0.8 | VA, HGIN |
| 1239 | 65 | female | + | N | N | Rectum | 0.1-0.4 | TA, HGIN |
| 1245 | 56 | female | + | Y | N | Rectum | 2 | VA, TA, HGIN |
| 1279 | 76 | male | + | Y | N | Rectum | 0.5-3.5 | VA, HGIN |
| 1299 | 48 | male | − | N | N | TC | 1.5 | TA, HGIN |
| 1322 | 58 | male | + | Y | N | CR | 0.2-2 | VA, TA, HGIN |
| 1357 | 55 | female | − | N | N | HF | 1 | TA |
| 1362 | 56 | female | + | N | N | Rectum | NA | HGIN |
| 2077 | 68 | male | − | N | N | CR | 1 | MA |
| 2115 | 53 | male | − | N | N | DC, SC | 0.7 | MA, HGIN |
| 2137 | 72 | female | − | Y | N | AC, TC | 1 | MA, VA |
| 2164 | 41 | male | + | Y | N | NA | NA | VA, TA, HGIN |
| 2167 | 76 | male | + | Y | N | LCR | 0.4-2 | MA, VA, HGIN |
| 2173 | 63 | male | + | N | N | Rectum | 0.3 | HGIN |
| 2195 | 48 | male | + | Y | N | Rectum | 0.1-0.2 | VA, HGIN |
| 2271 | 49 | male | − | N | N | SC | 1 | AP |
| 2435 | 48 | female | − | N | N | AC | 1.3 | AP |
| 3024 | 54 | male | − | N | N | Rectum | 1 | TA |
| 3025 | 49 | male | − | N | N | LCR | 1 | TA |
| 3028 | 58 | female | − | N | N | LCR | 0.8 | TA, HGIN |
| 3037 | 51 | female | + | Y | N | Rectum | 2 | VA, TA, HGIN |
| 3065 | 50 | male | − | N | Y | Rectum | 0.5 | SA, HGIN |
| 3069 | 49 | male | − | N | N | HF | 1 | MA, TA |
| 3071 | 51 | male | − | N | N | RCR | 0.6 | TA, HGIN |
| 3102 | 66 | male | − | Y | N | RCR, SF | 1.5 | MA, VA, TA |
| 3106 | 54 | male | − | N | N | NA | NA | MA, TA, HGIN |
| 3116 | 58 | male | − | N | N | AC | 1.3 | MA, TA, HGIN |
| 3133 | 59 | male | − | N | N | Rectum | 1 | TA |
| 3202 | 57 | male | + | Y | N | SC | NA | VA, TA |
| 3219 | 51 | female | + | Y | N | Rectum | 0.2-0.4 | VA, TA |
| 3245 | 52 | male | − | Y | N | NA | 1 | VA, TA, HGIN |
| 3281 | 58 | male | − | N | N | RCR | 1 | MA |

CR: colorectum; LCR and RCR: left-sided and right-sided colorectum; AC, TC, DC, and SC: ascending, transverse colon, descending, and sigmoid colon; AP: adenomatous polyp; MA, VA, TA, SA: multiple, villous, tubular, and serrated adenomas; HF and SF: hepatic and splenic flexure; HGIN: high-grade intraepithelial neoplasia;

Table S3. Performance characteristics of the sDNA test for the detection of CRC in three clinical sites.

| Hospital | Sensitivity % (n/N) | Specificity % (n/N) | Consistency rate* % (n/N) | Kappa |
| --- | --- | --- | --- | --- |
| The Sixth Affiliated Hospital of Sun Yat-sen University | 84.3 (113/134) | 97.3 (254/261) | 92.9 (367/395) | 0.84 |
| Nanfang Hospital of Southern Medical University | 82.8 (101/122) | 98.3 (232/236) | 93.0 (333/358) | 0.84 |
| Shandong Cancer Hospital and Institute | 84.4 (87/103) | 98.6 (213/216) | 94.0 (300/319) | 0.86 |
| Total | 83.8 (301/359) | 98.0 (699/713) | 93.3 (1000/1072) | 0.84 |

*The consistency rate is defined as the number of true positives plus the number of true negatives divided by the total number of cases.

Table S4. Results of the sDNA test in postoperative patients with CRC.

| Patient ID | Age | Gender | Before tumor resection (Positive/Negative) | After tumor resection (Positive/Negative) |
| --- | --- | --- | --- | --- |
| 1014 | 61 | Male | Positive | Negative |
| 1103 | 48 | Male | Positive | Negative |
| 1104 | 45 | Male | Positive | Negative |
| 1121 | 47 | Male | Positive | Negative |
| 1123 | 45 | Male | Positive | Negative |
| 1124 | 65 | Male | Positive | Excluded (ACTB CT value invalid) |
| 1134 | 64 | Male | Positive | Negative |
| 1143 | 60 | Male | Positive | Negative |
| 1145 | 67 | Male | Positive | Negative |
| 1148 | 67 | Female | Positive | Negative |
| 1150 | 75 | Male | Positive | Negative |
| 1154 | 48 | Female | Positive | Negative |
| 1157 | 58 | Male | Positive | Negative |
| 1162 | 60 | Male | Positive | Negative |
| 1163 | 63 | Female | Positive | Negative |
| 1164 | 65 | Male | Positive | Negative |
| 1166 | 58 | Female | Positive | Negative |
| 1170 | 69 | Male | Positive | Negative |
| 1173 | 48 | Female | Positive | Negative |
| 1177 | 49 | Male | Positive | Negative |
| 1180 | 53 | Female | Positive | Negative |
| 1181 | 46 | Female | Positive | Negative |
| 1188 | 41 | Male | Negative | Negative |
| 1201 | 58 | Female | Positive | Negative |
| 1207 | 45 | Female | Positive | Negative |
| 3185 | 63 | Female | Positive | Negative |
| 3348 | 51 | Female | Positive | Negative |
| 3176 | 62 | Female | Positive | Negative |
| 3179 | 64 | Male | Positive | Negative |
| 3242 | 68 | Female | Positive | Negative |
| 3304 | 54 | Female | Positive | Negative |
| 3164 | 50 | Female | Positive | Negative |
| 3305 | 42 | Female | Positive | Negative |
| 3118 | 52 | Male | Positive | Negative |
| 3335 | 66 | Male | Positive | Negative |
| 3308 | 49 | Male | Positive | Negative |
| 3310 | 67 | Male | Positive | Negative |
| 3209 | 51 | Male | Negative | Negative |
| 3204 | 46 | Female | Positive | Negative |
| 3228 | 71 | Male | Positive | Negative |

Table S5. The effect of interfering diseases on the outcome of the sDNA test.

| Disease types and distribution | | | Number | Number of positive | Number of negative |
| --- | --- | --- | --- | --- | --- |
| Interfering diseases | Cancers and other disorders in the digestive tracts | Inflammatory bowel disease | 1 | 0 | 1 |
|  |  | Gastritis | 11 | 1 | 10 |
|  |  | Gastric cancer | 11 | 0 | 11 |
|  |  | Esophagitis | 8 | 0 | 8 |
|  |  | Esophageal cancer | 12 | 0 | 12 |
|  |  | Esophageal mucosa roughness | 1 | 0 | 1 |
|  |  | Laryngeal cancer | 1 | 0 | 1 |
|  |  | Pancreatic cancer | 3 | 0 | 3 |
|  |  | Liver cancer | 1 | 0 | 1 |
|  |  | Biliary cancer | 1 | 0 | 1 |
|  |  | Duodenal cancer | 1 | 0 | 1 |
|  | Cancers and other disorders not in the digestive tracts | Rheumatoid arthritis | 8 | 0 | 8 |
|  |  | Lung cancer | 6 | 1 | 5 |
|  |  | Lung squamous carcinoma | 8 | 0 | 8 |
|  |  | Prostate cancer | 10 | 0 | 10 |
|  |  | Cholangiocarcinoma | 4 | 0 | 4 |
|  |  | Cervical squamous carcinoma | 1 | 0 | 1 |
|  |  | Cervical cancer | 1 | 0 | 1 |
|  |  | Breast cancer | 4 | 0 | 4 |
|  |  | Microbial infection | 10 | 0 | 10 |
| Total | | | 103 | 2 | 101 |


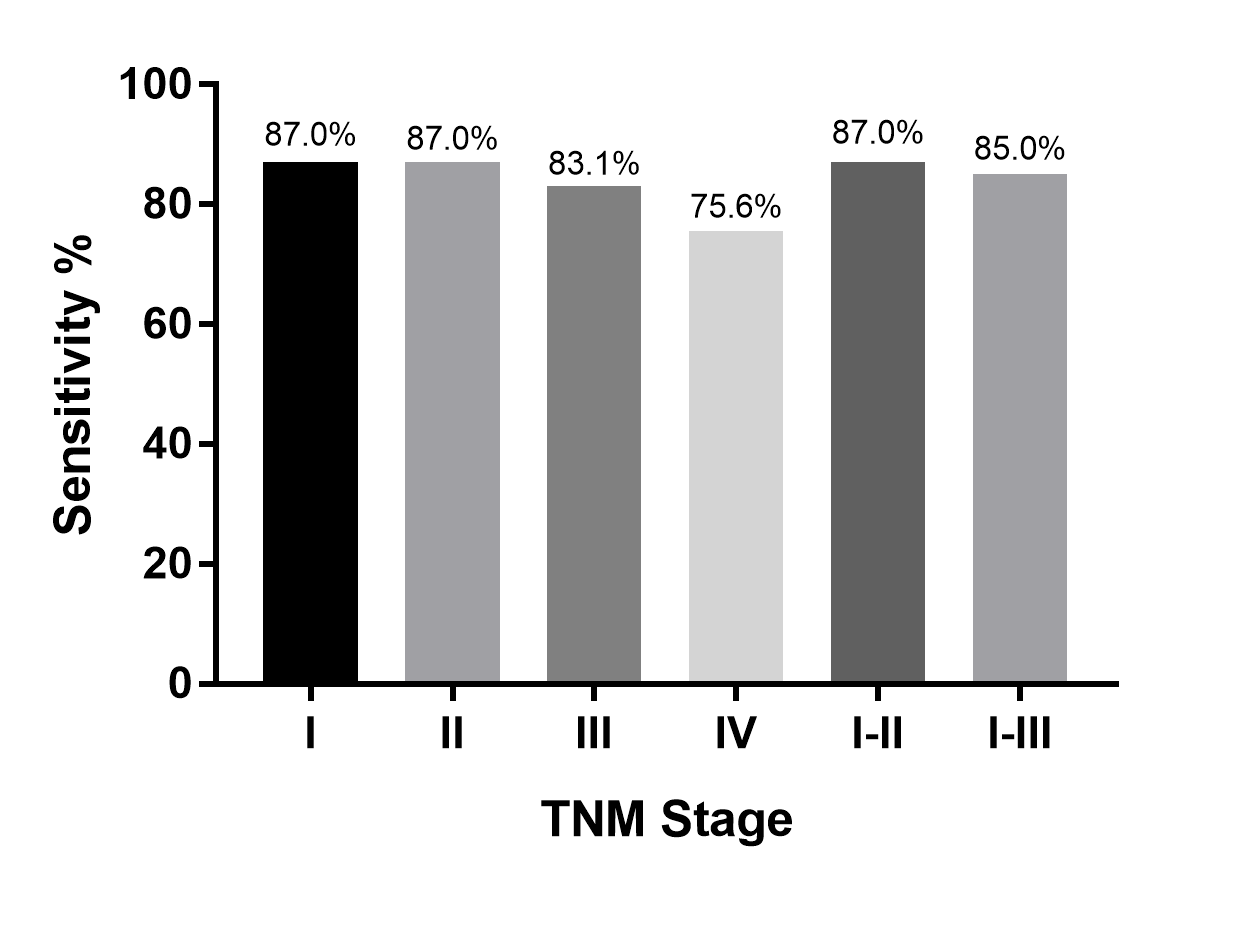


Figure S1. Sensitivity of the sDNA test for distinct TNM stages of CRC.
